# Genetic and non-genetic factors influencing milk composition and cow metabolism

**DOI:** 10.5194/aab-69-541-2026

**Published:** 2026-10-02

**Authors:** Jindřich Čítek, Eva Samková, Michaela Brzáková, Oto Hanuš, Libor Večerek, Eva Jozová, Lucie Hasoňová, Eva Baldíková, Sulaiman Mohammed Iliyasu, Michael Rost

**Affiliations:** 1 Faculty of Agriculture and Technology, University of South Bohemia, 370 05 České Budějovice, Czech Republic; 2 Institute of Animal Science, 104 00 Praha-Uhříněves, Czech Republic; 3 Dairy Research Institute, Ltd., 160 00 Praha 6, Czech Republic

## Abstract

The aim of this study was to evaluate the influence of genetic and non-genetic factors on milk composition and cow metabolism. Czech Holstein ( n=230 ) and Czech Simmental cows ( n=289 ) were analysed. The effects of farm, breed, parity, lactation stage, and polymorphisms on the *AGPAT6*, *SCD1*, *FASN*, *LEP*, and *DGAT1 *genes were evaluated. The contents of protein, fat, and fatty acids (FAs); the levels of β -hydroxybutyric acid (BHB), citric acid, acetone, and urea; and somatic cell scores were analysed. The farm and lactation stages were the most significant factors influencing the parameters, as well as their interactions. The effects of parity and breed were much weaker. The impact of gene polymorphisms was very low, as were the interactions between gene and farm, between lactation stage and farm, and between parity and lactation stage. BHB levels were influenced by farm in the models including all genes and by parity in the models including *AGPAT6*, *SCD1*, and *DGAT1* genes. In contrast, the effect of lactation stage on BHB levels was not significant in any model. The contents of protein and lactose were influenced by parity and lactation stage and, in some cases, by farm–lactation stage interactions and by gene polymorphisms. Urea, citric acid, acetone, and FA levels were affected by the lactation stage, and urea was also affected by the interaction of the farm and lactation stage. Daily milk production correlated negatively and significantly with components other than urea, acetone, BHB, and polyunsaturated FAs. The urea content and milk yield were significantly positively correlated. The correlations of BHB with fat / protein and fat / lactose ratios were significantly positive, but the prediction of ketosis in individual cows does not seem to be reliable. The level of BHB in milk, which is routinely assessed during milk performance testing, is suggested as a field predictor of clinical or subclinical ketosis in animals.

## 1 Introduction

Milk production has a significant impact on the metabolism of dairy cows. The enormous demands for energy, protein, and other essential nutrients places a considerable burden on the organism, particularly during early lactation. In this context, the most important metabolic disorder in dairy cows is postpartum ketonaemia, characterized by elevated serum beta-hydroxybutyrate (BHB) concentrations (Pothmann et al., 2026). It primarily affects high-producing cows at the beginning of lactation (Kučević et al., 2026). Both studies examined the associations among BHB, indicators of milk performance, and health status.

Ketone bodies (BHB, acetone) play a crucial role in energy metabolism. Ketosis is one of the most common metabolic disorders in dairy cows, with an incidence ranging from 40 % to 80 %. Other metabolites have also been investigated, e.g. milk urea nitrogen (Zhao et al., 2025). Several recent studies investigating cow metabolism have described associations between blood and milk parameters (Andjelić et al., 2022; Konečný et al., 2026; James et al., 2024). Magro et al. (2025) reported correlations between the milk fat-to-protein ratio (FPR) and the concentrations of BHB, non-esterified fatty acids (NEFAs), and cholesterol.

Compared with other cows, the progeny of the best sires showed a lower incidence of hyperketonaemia. The authors concluded that identifying animals with high and low genetic merit for metabolic traits is a valuable approach. A total of 42 fatty acids (FAs) and milk quality measurements were found to be associated with feed efficiency (James et al., 2024). Bezdíček et al. (2024) reported that mobilization of body fat reserves during negative energy balance (NEB) in early lactation is associated with increased blood FA concentrations. When a critical threshold is exceeded, both reproduction and health may be adversely affected.

The synthesis of FAs plays a critical role in shaping milk production characteristics in dairy cattle (Ardicli et al., 2024). The authors examined the impact of specific genes within the FA metabolic pathway. The individual effects of the fatty acid-binding protein (*FABP3*), lactotransferrin (*LTF*), and annexin A9 (*ANXA9*) genes significantly influenced both milk yield and fat yield. These genes identified a haplotype that may serve as a robust genetic marker for both fat yield and percentage. Significant associations were also found among milk FA composition and polymorphisms in genes of fatty acid synthase (*FASN*), peroxisome proliferator-activated receptor gamma coactivator 1-alpha (*PPARGC1A*), ATP-binding cassette subfamily G member 2 (*ABCG2*), and insulin-like growth factor 1 (*IGF1*) (Li et al., 2016; Mauric et al., 2019). In addition, polymorphisms in the stearoyl-CoA desaturase 1 (*SCD1*) gene contributed to the phenotypic variation in milk FA composition, indicating their potential use in dairy cattle breeding programmes (Safina et al., 2018).

On the other hand, no significant associations were found between polymorphisms in the 1-acylglycerol-3-phosphate O-acyltransferase 6 (*AGPAT6*) gene and milk FA composition. However, the GC dinucleotide allele of the diacylglycerol O-acyltransferase 1 (*DGAT1*) gene was associated with lower proportions of palmitic acid (C16:0) and higher proportions of oleic acid (C18:1 *cis*-9) and linoleic acid (C18:2 *cis*-9, *cis*-12), as well as a higher elongation index. Accordingly, the GC dinucleotide allele was associated with lower milk fat content and short-chain FA proportions and higher monounsaturated FA and polyunsaturated FA proportions (Nafikov et al., 2014).

Recently, a genomic approach has been employed to investigate the genetic regulation of FAs (Freitas et al., 2020). The authors reported moderate heritabilities for different groups of FAs, ranging from 0.24 to 0.47. Ghavi Hossein-Zadeh (2021) reported broader ranges for FA groups and individual FAs, ranging from 0.092 to 0.428 and from 0.109 to 0.421, respectively. The application of genotype imputation, i.e. the use of haplotype information obtained from microarrays with a high number of single-nucleotide polymorphisms (SNPs) in animals genotyped for a lower number of SNPs, represents a valuable approach in genomic analyses (Klímová et al., 2020). More recently, increasingly complex approaches have been used to investigate genetic regulation in agricultural animals (Glazko et al., 2025), with particular attention being paid to interactions among animals, microbiomes, and viromes (Glazko et al., 2021).

In this study, milk components, including FAs, were analysed. Particular attention was paid to somatic cell count (SCC) and minor milk constituents associated with the metabolic status of dairy cows, namely, lactose, urea, citric acid, acetone, and BHB. The effects of the farm, breed, parity, and lactation stage were evaluated. As in previous studies, the effects of several major genes on milk composition, polymorphisms in the *AGPAT6*, *SCD1*, *FASN*, *LEP* (leptin), and *DGAT1* genes were also included to evaluate their potential influence on indicators of metabolic status. Furthermore, the interactions between polymorphism and farm, polymorphism and lactation stage, farm and lactation stage, and parity and lactation stage were examined. Correlations among milk components were determined, and their potential for evaluating metabolic status was discussed. The principal objective was to assess the factors influencing milk composition and the metabolic status under field conditions.

## 2 Materials and methods

### 2.1 Animals

All animal experiments were conducted under the supervision of the Institutional Animal Care and Use Committee of the Faculty of Agriculture and Technology, University of South Bohemia in České Budějovice (USB), with approval no. 22036/2019-MZE-18134. DNA was extracted non-invasively from the milk samples.

A total of 519 animals were involved in the study, comprising Czech Holstein ( n=230 ) and Czech Simmental ( n=289 ) cows. They were fathered by 122 sires.

The cows were housed in five farms in free housing. Farm 1 contributed 247 Holstein records, whereas farms 3, 4, and 5 contributed 95, 247, and 91 Czech Simmental records, respectively. Farm 2 included both breeds and contributed 116 Holstein and 18 Czech Simmental records. The milk samples were obtained during the first ( n=693 ), second ( n=87 ), and third or higher lactations ( n=34 ) throughout the year. Approximately half of the cows were sampled twice ( n=295 ), and the other half were sampled only once ( n=224 ), each time within the same ongoing lactation period. The feeding diet was a total mixed ratio (TMR), consisting of maize silage, grass silage, hay, and feed concentrate throughout the year. After accounting for repeated measurements from individual cows, the final dataset included 814 records.

### 2.2 Sampling and milk analyses

The individual milk samples were collected only during the regular testing of milk performance and quality. After sampling, the samples were immediately transported under cold conditions to the laboratory and analysed. No chemical preservatives were used in any of the samples.

The milk composition, i.e. the contents of fat, crude protein, lactose monohydrate, urea, citric acid, acetone, BHB, SCC, and FAs (g 100 g^−1^ milk), was determined in an accredited laboratory of the Czech–Moravian Breeders Association (ČSN EN ISO/IEC 17025, The Czech Office for Standards, Metrology and Testing, 2018) using Fourier transform mid-infrared spectroscopy (FT-MIR) (ČSN EN ISO 13366-1 (570531), Czech Normalization Institute, 2008; ČSN EN ISO 13366-2 (570531), Czech Normalization Institute, 2007; Samková et al., 2020). Foss Electric (Hilleroed, Denmark) and Bentley Instruments (Chaska, MN, USA) were used as suppliers of the instruments. The SCC values were log_10_-transformed to normalize their distribution. The somatic cell score (SCS) was calculated according to the following equation:

1 $$\mathrm{SCS}={\mathrm{log}}_{10}\left(\mathrm{SCC}\right)+3.$$

The instruments underwent proficiency testing, with consistently successful results. The extended result uncertainties (95 % probability level) were ±2.77 % for fat (0.101 % for the original unit as a gram per 100 grams), ±2.59 % for crude (total nitrogen content × 6.38) protein (0.085 %), ±2.77 % for lactose monohydrate (0.115 %), and ±9.3 % for SCC < 900 × 10^3^ mL^−1^.

The descriptive statistics of milk performance and the contents of components on the sampling day are given in Table 1.

**Table 1:** Data on daily milk yield and milk parameters (all-day) on collection Day 1 (first sampling) and Day 2 (second sampling), obtained from the performance testing.

Traits	First sampling					Second sampling				
	n	Mean	SD	Min.	Max.	n	Mean	SD	Min.	Max.
Parity	429	1.15	0.44	1	3	385	1.24	0.53	1	3
Days in milk	429	160	81	8	303	385	157	87	6	304
Milk yield and milk parameters										
Milk yield (kg)	433	26.2	7.66	3.3	54.3	391	27.1	8.55	9.5	56.5
Fat (g 100 g^−1^)	428	4.29	0.71	2.15	8.48	388	4.37	0.74	2.58	9.85
Protein (g 100 g^−1^)	428	3.53	0.33	2.03	4.95	388	3.61	0.34	2.70	4.99
Lactose (g 100 g^−1^)	428	5.14	0.18	3.62	5.51	388	5.13	0.20	4.27	5.52
Fat-to-protein ratio	428	1.22	0.19	0.56	2.25	388	1.22	0.22	0.75	3.22
Fat-to-lactose ratio	428	0.84	0.15	0.44	1.74	388	0.85	0.16	0.52	2.08
SCS (log)	378	4.992	0.416	4.204	6.418	293	5.011	0.472	3.845	6.828
Urea (mg 100 mL^−1^)	425	24.09	7.50	9.90	88.4	213	29.53	6.19	10.10	44.80
Citric acid (%)	423	0.20	0.02	0.14	0.28	172	0.19	0.02	0.15	0.27
Acetone (mmol L^−1^)	423	0.99	0.06	0.01	0.36	172	0.14	0.07	0.01	0.40
BHB (mmol L^−1^)	423	0.03	0.03	0.01	0.20	172	0.06	0.03	0.01	0.19
FFAs (mmol 100 g^−1^ fat)	414	0.99	0.39	0.09	2.23	168	1.08	0.54	0.04	2.36
Fatty acids (g 100 g^−1^ milk)										
Palmitic acid	424	1.51	0.31	0.70	3.26	171	1.45	0.23	0.85	2.24
Stearic acid	424	0.57	0.11	0.28	1.02	171	0.55	0.11	0.21	0.98
Oleic acid	424	1.17	0.23	0.46	2.08	171	1.15	0.24	0.22	2.02
Short-chain FAs	426	0.43	0.11	0.12	0.97	171	0.44	0.09	0.07	0.74
Medium-chain FAs	424	1.93	0.55	0.85	4.78	171	1.79	0.42	1.02	3.35
Long-chain FAs	424	1.50	0.32	0.52	2.90	171	1.46	0.33	0.43	2.79
Saturated FAs	426	2.91	0.60	1.37	6.24	171	2.85	0.46	1.41	4.50
Monounsaturated FAs	424	1.37	0.23	0.59	2.27	171	1.35	0.24	0.33	2.22
Polyunsaturated FAs	424	0.33	0.03	0.23	0.42	171	0.32	0.04	0.15	0.43
Trans FAs	426	0.12	0.02	0.01	0.18	171	0.11	0.02	0.04	0.16

n – number of samples; SD – standard deviation; SCS – somatic cell score = log_10_(somatic cell count) + 3; BHB – β -hydroxybutyric acid; FFAs – free fatty acids; FAs – fatty acids.

### 2.3 Genotyping

DNA was isolated from the milk samples using a MagCore HF16 Plus DNA/RNA extractor (RBC Bioscience, New Taipei, Taiwan). Genotyping was performed by the following the PCR/RFLP method.

*AGPAT6* gene alleles C and T were genotyped using fragment analysis as described by Littlejohn et al. (2014). Primer sequences were AGPAT6_5'-FAM-CAAGGC GGCGTAGACAAA – 3' and AGPAT6_R 5'- AGCCCCGTCAGAGGTTCAT – 3'. *SCD1* gene allele (c.878T > C) genotyping was performed using the PCR primers SCD_F_5`-ACCTGGCTGGTGAATAGTGCT-3' and SCD_R_5'-TCTGGCACGTAACCTAATACCCT-3'. Amplificat was digested by restriction enzyme *Fnu*4HI (Inostroza et al., 2013). *FASN* gene g.17924A > G SNP genotyping was performed using the primers FASN_F 5'- TGCTGTCCATGCGGGAAATC-3' and FASN_R 5'- TGAGCACATCTCGAAAGCCA-3'. PCR products were digested by the restriction enzyme *Msc*I as described previously (Cancino-Baier et al., 2021; Inostroza et al., 2013; Roy et al., 2006). *LEP* gene alleles M and W were genotyped using the primers LEP_F 5'GGGAAGGGCAGAAAGATAG and LEP_R 3' TGGCAGACTGTTGAGGATC. PCR products were digested by the restriction enzyme *Hph*I (Buchanan et al., 2002; Haegeman et al., 2000). *DGAT1* gene alleles A (alanine) and K (lysine), with the GC → AA exchange at positions 10433/10434, were genotyped using the primers DGAT1_F 5'-GCACCATCCTCTTCCTCAAG and DGAT1_R 3'- GGGGGCGAAGAGGAAGTAGTAG. PCR products were digested with the restriction enzyme *Cfr*10I (Kühn et al., 2004). Detailed information on the genotyping is in the Supplement. Genotype and allele frequencies and deviation from the Hardy–Weinberg equilibrium (HWE) of all analysed loci are presented in Table S1 in the Supplement.

### 2.4 Statistical analysis

Statistical analyses were performed using SAS 9.4 (SAS Institute, Cary, NC, USA). The dataset contained repeated measurements of milk components obtained from the first to sixth lactations. All test-day milk records collected between 1 and 305 d in milk (DIM) were included in the analysis. Individual test-day milk samples were analysed rather than whole-lactation records. To account for physiological changes during lactation, lactation stage was included as a fixed effect in the statistical model. To analyse the effects of the polymorphisms and other effects on milk components, a linear mixed model (MIXED procedure of the SAS system with repeated measurements) and the least-squares mean (LSM) method were used. The analysis was based on an unbalanced dataset, where some cows had missing trait measurements, and not all fixed effects were observed in each record. In accordance with the default setting of the MIXED procedure in SAS, only complete observations were used in the analysis. The model was developed as follows:

2 $$\begin{array}{rl} Y_{ijklmno} & =\mu +{\mathrm{gene}}_{i}+{\mathrm{farm}}_{j}+{\mathrm{lac}}_{k}+{\mathrm{lacstage}}_{l}\\ & +{\mathrm{breed}}_{m}+{\mathrm{sire}}_{n}+{\mathrm{cow}}_{o}+e_{ijklmno}, \end{array}$$

where $Y_{ijklmno}$ is the analysed milk component; gene_*i*_ is the fixed effect of the gene (class effect i=1 , 2, 3); farm_*j*_ is the fixed effect of the farm (class effect j=1 , …, 5); lac_*k*_ is the fixed effect of the parity (class effect k=1 , 2, ≥3 representing lactation order); lacstage_*l*_ is the fixed effect of the lactation stage (class effect l=1 , 2, 3 for days in milk 1–99, 100–200, 201–305), where records with DIM > 305 d were excluded during data editing because only standardized 305 d lactations were considered; breed_*m*_ is the fixed effect of the breed (class effect m=1 , 2 representing Czech Holstein cows and Czech Simmental cows); sire_*n*_ is the random effect of the sire of the cow; cow_*o*_ is the permanent environment of the cow (repeated measurement); and $e_{ijklmno}$ is the random residual effect.

The interactions of genotype–farm, genotype–lactation stage, farm–lactation stage, and parity–lactation stage were tested by including two-way interaction terms as fixed effects in the model. Post hoc comparisons were performed using Scheffé's test. Correlations between variables were computed using Pearson's correlation coefficient in SAS (PROC CORR procedure).

## 3 Results and discussion

Genotype and allele frequencies for all analysed loci are presented in the Supplement (Table S1). The *LEP*, *SCD1*, *FASN*, and *AGPAT6 *polymorphisms deviated significantly from Hardy–Weinberg equilibrium (HWE), whereas the *DGAT1* polymorphism conformed to HWE. Such deviations may arise from selection pressure, non-random mating, and population structure, which are common features of managed livestock populations (Banos et al., 2008).

The farm and lactation stage were the most significant factors influencing the content of milk components, as well as their interactions (Tables 2–6). The effect of the lactation stage was also found to be significant by other authors (Reus et al., 2023). The impact of parity and breed was much weaker. One possible explanation is the partial overlap between breed and farm effects as most farms kept only one breed. In contrast, the significance of parity increased after inclusion of the lactation stage, likely owing to improved adjustment for lactation-related variability.

**Table 2:** Significance of effects on daily milk yield and milk parameters ( p -values) – *AGPAT6* gene, significant values in bold.

Traits	n	Farm (F)	Gene (G)	Lactation		Breed (B)	Interaction			
				Parity (P)	Stage (S)		G × F	G × S	F × S	P × S
Milk yield and milk parameters										
Milk yield (kg)	787	**0.0001**	0.4556	< **0.001**	< **0.001**	**0.0061**	0.8544	0.1155	**0.0086**	**0.0006**
Fat (g 100 g^−1^)	746	< **0.001**	0.8972	**0.0111**	< **0.001**	0.3971	0.9940	0.1984	**0.0085**	0.1687
Protein (g 100 g^−1^)	746	< **0.001**	0.9011	**0.0113**	< **0.001**	0.4034	0.3285	0.2849	< **0.001**	0.7678
Lactose (g 100 g^−1^)	746	0.1682	0.2700	**0.0004**	< **0.001**	0.9455	0.5436	0.3553	**0.0411**	0.5228
SCS (log)	746	< **0.001**	0.9025	0.0674	**0.0031**	0.9559	0.8179	**0.0176**	0.1942	0.3757
Urea (mg 100 mL^−1^)	630	< **0.001**	0.1481	0.8496	**0.0010**	0.9615	0.5126	0.0890	< **0.001**	0.7709
Citric acid (%)	630	< **0.001**	0.1597	0.1135	**0.0094**	**0.0322**	0.7655	0.8165	0.8377	0.3048
Acetone (mmol L^−1^)	630	< **0.001**	0.3102	0.3542	< **0.001**	0.3033	0.4272	**0.0019**	0.3901	0.1807
BHB (mmol L^−1^)	630	< **0.001**	0.1284	**0.0243**	0.5002	0.0580	0.7315	**0.0101**	0.2406	**0.0398**
FFAs (mmol 100 g^−1^ fat)	616	< **0.001**	0.8904	0.1287	< **0.001**	0.7896	0.8920	0.3923	0.6127	0.6066
Fatty acids (g 100 g^−1^ milk)										
Palmitic acid	630	< **0.001**	0.8716	0.0556	< **0.001**	0.2447	0.9938	0.4372	**0.0101**	0.0843
Stearic acid	630	0.1664	0.7164	0.2361	< **0.001**	0.1119	0.9996	0.0918	0.2353	0.6200
Oleic acid	630	< **0.001**	0.9990	0.6300	< **0.001**	0.0889	0.9916	0.1480	**0.0041**	0.7987
Short-chain FAs	629	< **0.001**	0.8894	**0.0183**	**0.0005**	0.0766	N/A	0.2867	**0.0436**	0.3307
Medium-chain FAs	630	< **0.001**	0.5036	0.1345	< **0.001**	0.1502	0.7013	0.3367	**0.0003**	**0.0429**
Long-chain FAs	630	**0.0003**	0.9472	0.4729	< **0.001**	0.1428	N/A	0.1134	**0.0240**	0.9355
Saturated FAs	630	< **0.001**	0.8621	**0.0173**	< **0.001**	0.2529	0.9987	0.2811	**0.0189**	0.1246
Monounsaturated FAs	630	< **0.001**	0.9966	0.7500	< **0.001**	**0.0421**	N/A	0.1303	**0.0030**	0.7983
Polyunsaturated FAs	630	0.0618	0.7391	0.1894	< **0.001**	**0.0128**	0.5153	0.3496	0.1964	0.8757
*Trans* FAs	627	0.2142	**0.0355**	< **0.001**	**0.0482**	**0.0289**	0.8719	0.1642	0.3021	0.8654

n – number of samples; N/A – not applicable due to the low number of samples; SCS – somatic cell score = log_10_ (somatic cell count) + 3; BHB – β -hydroxybutyric acid; FFAs – free fatty acids; FAs – fatty acids.

**Table 3:** Significance of effects on daily milk yield and milk parameters ( p -values) – *SCD1* gene, significant values in bold.

Traits	n	Farm (F)	Gene (G)	Lactation		Breed (B)	Interaction			
				Parity (P)	Stage (S)		G × F	G × S	F × S	P × S
Milk yield and milk parameters										
Milk yield (kg)	810	< **0.001**	0.8591	< **0.001**	< **0.001**	**0.0208**	0.1438	0.0533	**0.0132**	**0.0024**
Fat (g 100 g^−1^)	764	< **0.001**	0.6306	**0.0104**	< **0.001**	0.2651	0.9941	0.8944	**0.0038**	0.1761
Protein (g 100 g^−1^)	764	**0.0143**	0.6160	**0.0017**	< **0.001**	**0.0061**	0.9767	0.7467	**0.0007**	0.7955
Lactose (g 100 g^−1^)	764	0.1854	**0.0171**	< **0.001**	< **0.001**	0.6013	**0.0233**	0.0738	**0.0409**	0.7940
SCS (log)	764	< **0.001**	0.3426	**0.0346**	**0.0045**	0.8081	0.3568	0.5386	0.2268	0.2726
Urea (mg 100 mL^−1^)	644	< **0.001**	**0.0321**	0.7485	**0.0022**	0.9874	0.8658	0.3330	< **0.001**	0.7698
Citric acid (%)	644	< **0.001**	**0.0427**	0.1884	**0.0043**	0.1402	0.7128	0.9754	0.8483	0.5741
Acetone (mmol L^−1^)	644	< **0.001**	0.4414	0.3830	< **0.001**	0.4024	0.7816	0.9227	0.3548	0.1897
BHB (mmol L^−1^)	644	< **0.001**	0.2802	**0.0370**	0.3670	0.0817	0.8316	0.7203	0.2465	**0.0499**
FFAs (mmol 100 g^−1^ fat)	629	< **0.001**	**0.0361**	0.1359	< **0.001**	0.7007	0.0849	0.7041	0.5474	0.5825
Fatty acids (g 100 g^−1^ milk)										
Palmitic acid	644	< **0.001**	0.6143	**0.0327**	< **0.001**	0.1536	0.9940	0.8059	**0.0034**	0.0546
Stearic acid	644	0.3127	0.2589	0.3457	< **0.001**	**0.0372**	0.6306	0.9825	0.1265	0.8237
Oleic acid	644	< **0.001**	0.1081	0.5888	< **0.001**	**0.0115**	0.7437	0.9611	**0.0012**	0.7259
Short-chain FAs	643	< **0.001**	0.4690	**0.0158**	**0.0006**	0.0918	0.9999	0.9895	**0.0319**	0.2748
Medium-chain FAs	644	< **0.001**	0.7459	0.0805	< **0.001**	0.1325	0.9678	0.3596	**0.0001**	0.0557
Long-chain FAs	644	**0.0003**	0.1740	0.5161	< **0.001**	**0.0274**	0.7283	0.9446	**0.0117**	0.9312
Saturated FAs	644	< **0.001**	0.6584	**0.0115**	< **0.001**	0.1851	0.9999	0.8450	**0.0087**	0.1102
Monounsaturated FAs	644	< **0.001**	0.1456	0.7221	< **0.001**	**0.0051**	0.8182	0.9836	**0.0007**	0.7500
Polyunsaturated FAs	644	0.0670	0.0819	0.1947	< **0.001**	**0.0030**	0.8733	0.7404	0.1644	0.9861
*Trans* FAs	641	0.3152	0.2343	< **0.001**	**0.0262**	0.1853	0.8975	0.3858	0.2612	0.8481

n – number of samples; SCS – somatic cell score = log_10_(somatic cell count) + 3; BHB – β -hydroxybutyric acid; FFAs – free fatty acids; FAs – fatty acids.

**Table 4:** Significance of effects on daily milk yield and milk parameters ( p values) – *FASN* gene; significant values in bold.

Traits	n	Farm (F)	Gene (G)	Lactation		Breed (B)	Interaction			
				Parity (P)	Stage (S)		G × F	G × S	F × S	P × S
Milk yield and milk parameters										
Milk yield (kg)	811	< **0.001**	0.1547	< **0.001**	< **0.001**	**0.0141**	0.3089	0.2959	**0.0237**	**0.0037**
Fat (g 100 g^−1^)	765	< **0.001**	0.3210	**0.0090**	< **0.001**	0.2527	0.4122	0.4206	**0.0060**	0.1936
Protein (g 100 g^−1^)	765	**0.0191**	0.0946	**0.0005**	< **0.001**	**0.0037**	**0.0333**	0.8310	**0.0025**	0.8378
Lactose (g 100 g^−1^)	765	0.1682	0.6842	< **0.001**	< **0.001**	0.8010	0.6435	0.7946	**0.0383**	0.7934
SCS (log)	765	< **0.001**	0.3209	**0.0323**	**0.0068**	0.9549	0.6872	0.9508	0.2843	0.2710
Urea (mg 100 mL^−1^)	644	< **0.001**	**0.0252**	0.6118	**0.0012**	0.8938	0.5670	0.4225	< **0.001**	0.6151
Citric acid (%)	644	< **0.001**	0.6179	0.1564	**0.0045**	0.0950	0.7460	0.2678	0.8116	0.4852
Acetone (mmol L^−1^)	644	< **0.001**	0.8451	0.4560	< **0.001**	0.3200	0.9813	0.6746	0.3729	0.1847
BHB (mmol L^−1^)	644	< **0.001**	0.7948	0.0510	0.3601	0.0751	0.9434	0.1806	0.2711	**0.0368**
FFAs (mmol 100 g^−1^ fat)	629	< **0.001**	0.7238	0.1648	< **0.001**	0.8589	0.2460	0.2990	0.5550	0.7338
Fatty acids (g 100 g^−1^ milk)										
Palmitic acid	644	< **0.001**	0.0985	**0.0283**	< **0.001**	0.1233	0.3908	0.6159	**0.0060**	0.0527
Stearic acid	644	0.1851	0.3840	0.3206	< **0.001**	**0.0350**	0.1336	0.0826	0.1468	0.8122
Oleic acid	644	< **0.001**	0.4963	0.5539	< **0.001**	**0.0147**	0.0656	0.1126	**0.0013**	0.7895
Short-chain FAs	643	< **0.001**	0.2647	**0.0125**	**0.0010**	0.0566	0.5330	0.8702	**0.0452**	0.2632
Medium-chain FAs	644	< **0.001**	0.1613	0.0683	< **0.001**	0.0942	0.4556	0.2710	**0.0003**	0.0535
Long-chain FAs	644	**0.0002**	0.4849	0.4861	< **0.001**	**0.0322**	0.1266	0.1080	**0.0129**	0.9432
Saturated FAs	644	< **0.001**	0.1217	**0.0101**	< **0.001**	0.1442	0.4765	0.6646	**0.0151**	0.1055
Monounsaturated FAs	644	< **0.001**	0.3588	0.6888	< **0.001**	**0.0064**	0.0842	0.1073	**0.0011**	0.7964
Polyunsaturated FAs	644	**0.0407**	0.2876	0.2341	< **0.001**	**0.0029**	0.2661	0.1986	0.1980	0.9885
*Trans* FAs	641	0.4249	0.6541	< **0.001**	**0.0469**	0.1749	0.3372	0.4634	0.2435	0.8100

n – number of samples; SCS – somatic cell score = log_10_(somatic cell count) + 3; BHB – β -hydroxybutyric acid; FFAs – free fatty acids; FAs – fatty acids.

**Table 5:** Significance of effects on daily milk yield and milk parameters ( p -values) – *LEP* gene, significant values in bold.

Traits	n	Farm (F)	Gene (G)	Lactation		Breed (B)	Interaction			
				Parity (P)	Stage (S)		G × F	G × S	F × S	P × S
Milk yield and milk parameters										
Milk yield (kg)	675	**0.0018**	**0.0461**	< **0.001**	< **0.001**	**0.0098**	0.6023	0.4529	**0.0084**	**0.0019**
Fat (g 100 g^−1^)	646	< **0.001**	0.6561	**0.0204**	< **0.001**	0.3477	0.4818	0.7110	**0.0050**	0.0969
Protein (g 100 g^−1^)	646	**0.0133**	0.3947	**0.0087**	< **0.001**	**0.0041**	0.5154	0.9435	**0.0105**	0.8400
Lactose (g 100 g^−1^)	646	0.2496	0.7669	**0.0013**	< **0.001**	0.9699	0.0978	0.7056	**0.0241**	0.5214
SCS (log)	646	< **0.001**	0.3856	0.0883	**0.0184**	0.9372	0.4890	0.2542	**0.0805**	0.5454
Urea (mg 100 mL^−1^)	551	< **0.001**	**0.0318**	0.4689	< **0.001**	0.9695	0.8040	0.2276	< **0.001**	0.5567
Citric acid (%)	551	< **0.001**	0.1724	0.1869	**0.0008**	0.0783	0.2778	0.8687	0.5285	0.6224
Acetone (mmol L^−1^)	551	< **0.001**	0.7757	0.6757	< **0.001**	0.2722	0.7949	0.8586	0.7064	0.6140
BHB (mmol L^−1^)	551	< **0.001**	0.9700	0.1567	0.1435	0.0522	0.4991	0.7437	0.3095	0.2779
FFAs (mmol 100 g^−1^ fat)	536	< **0.001**	0.9046	0.2392	< **0.001**	0.7243	0.9384	0.2171	0.2447	0.6048
Fatty acids (g 100 g^−1^ milk)										
Palmitic acid	551	< **0.001**	0.5322	**0.0129**	< **0.001**	0.2077	0.6698	0.7199	**0.0240**	**0.0154**
Stearic acid	551	0.3569	0.5951	0.6215	< **0.001**	0.0580	0.3276	0.9678	**0.0430**	0.7772
Oleic acid	551	< **0.001**	0.9786	0.8236	< **0.001**	**0.0286**	0.9656	0.2137	**0.0004**	0.9068
Short-chain FAs	550	< **0.001**	0.6881	**0.0102**	**0.0016**	0.0977	0.3529	0.7382	**0.0394**	0.1822
Medium-chain FAs	551	< **0.001**	0.7515	**0.0390**	< **0.001**	0.1148	0.2364	0.8138	**0.0005**	**0.0092**
Long-chain FAs	551	**0.0002**	0.8744	0.8373	< **0.001**	**0.0324**	0.8498	0.5038	**0.0027**	0.9551
Saturated FAs	551	< **0.001**	0.6534	**0.0038**	< **0.001**	0.2519	0.5714	0.7908	**0.0377**	**0.0424**
Monounsaturated FAs	551	**0.0001**	0.9621	0.9381	< **0.001**	**0.0175**	0.9292	0.1318	**0.0003**	0.8711
Polyunsaturated FAs	551	0.1047	0.8606	0.2912	**0.0005**	**0.0048**	0.6314	0.2703	0.1444	0.9911
*Trans* FAs	548	0.1776	0.2518	< **0.001**	0.2427	0.1220	0.1576	0.2441	0.2531	0.7429

n – number of samples; SCS – somatic cell score = log_10_(somatic cell count) + 3; BHB – β -hydroxybutyric acid; FFAs – free fatty acids; FAs – fatty acids.

**Table 6:** Significance of effects on daily milk yield and milk parameters ( p -values) – *DGAT1* gene, significant values in bold.

Traits	n	Farm (F)	Gene (G)	Lactation		Breed (B)	Interaction			
				Parity (P)	Stage (S)		G × F	G × S	F × S	P × S
Milk yield and milk parameters										
Milk yield (kg)	813	**0.0001**	0.3100	< **0.001**	< **0.001**	**0.0139**	0.3238	0.1902	**0.0115**	**0.0024**
Fat (g 100 g^−1^)	767	< **0.001**	0.3120	**0.0085**	< **0.001**	0.3256	0.7430	0.8454	**0.0052**	0.1807
Protein (g 100 g^−1^)	767	**0.0171**	0.9347	**0.0016**	< **0.001**	**0.0045**	0.4159	0.0555	**0.0007**	0.7959
Lactose (g 100 g^−1^)	767	0.2989	0.0890	**0.0001**	< **0.001**	0.9378	**0.0317**	0.4617	**0.0307**	0.7668
SCS (log)	767	< **0.001**	**0.0043**	**0.0450**	**0.0065**	0.9944	0.2042	0.0933	0.3423	0.2110
Urea (mg 100 mL^−1^)	646	< **0.001**	0.7743	0.7682	**0.0017**	0.9244	0.8305	0.9944	< **0.001**	0.7626
Citric acid (%)	646	< **0.001**	0.4998	0.2292	**0.0049**	0.0714	0.4854	0.7670	0.8416	0.5295
Acetone (mmol L^−1^)	646	< **0.001**	0.8558	0.3979	< **0.001**	0.3137	0.8315	0.5727	0.3614	0.2052
BHB (mmol L^−1^)	646	< **0.001**	0.9383	**0.0355**	0.3636	0.0625	0.6144	0.7280	0.2196	0.0523
FFAs (mmol 100 g^−1^ fat)	631	< **0.001**	0.4651	0.1394	< **0.001**	0.8405	0.2555	0.5646	0.5567	0.6810
Fatty acids (g 100 g^−1^ milk)										
Palmitic acid	646	< **0.001**	0.3909	**0.0269**	< **0.001**	0.1898	0.6032	0.9512	**0.0050**	0.0551
Stearic acid	646	0.2848	0.8893	0.3323	< **0.001**	**0.0484**	0.9836	0.7209	0.1448	0.7911
Oleic acid	646	< **0.001**	0.7781	0.6046	< **0.001**	**0.0185**	0.9048	0.8546	**0.0011**	0.7981
Short-chain FAs	645	< **0.001**	0.3171	**0.0119**	**0.0005**	0.0840	0.7139	0.9089	**0.0386**	0.2808
Medium-chain FAs	646	< **0.001**	0.5279	**0.0694**	< **0.001**	0.1369	0.4117	0.7309	**0.0002**	0.0530
Long-chain FAs	646	**0.0002**	0.7930	0.5246	< **0.001**	**0.0400**	0.9214	0.7818	**0.0107**	0.9507
Saturated FAs	646	< **0.001**	0.3618	**0.0090**	< **0.001**	0.2125	0.6746	0.9715	**0.0130**	0.1104
Monounsaturated FAs	646	< **0.001**	0.7231	0.7327	< **0.001**	**0.0081**	0.8618	0.8092	**0.0007**	0.8219
Polyunsaturated FAs	646	0.0589	0.9430	0.2047	< **0.001**	**0.0040**	0.8855	0.2030	0.1513	0.9865
*Trans* FAs	643	0.3177	0.6494	< **0.001**	**0.0361**	0.1513	0.2197	0.7168	0.2270	0.8325

n – number of samples; SCS – somatic cell score = log_10_(somatic cell count) + 3; BHB – β -hydroxybutyric acid; FFAs – free fatty acids; FAs – fatty acids.

**Table 7:** Correlations ( p -value) among components of cow milk, significant values in bold.

Traits	n	Milk yield (kg)	n	Fat-to-protein ratio	n	Fat-to-lactose ratio	n	Fat (g 100 g^−1^)	n	Protein (g 100 g^−1^)	n	Lactose (g 100 g^−1^)
Protein (g 100 g^−1^)	814	- **0.406(** < **0.001)**	814	- **0.245(** < **0.001)**	814	**0.356(** < **0.001)**	816	**0.346(** < **0.001)**		–		
Lactose (g 100 g^−1^)	814	**0.142(** < **0.001)**	814	- **0.070(0.046)**	814	- **0.364(** < **0.001)**	816	- **0.161(** < **0.001)**	814	- **0.155(** < **0.001)**		–
Urea (mmol L^−1^)	638	**0.450(** < **0.001)**	638	**0.250(** < **0.001)**	638	**0.151(** < **0.001)**	638	**0.178(** < **0.001)**	638	- **0.089(0.024)**	638	0.060(0.129)
Citric acid (%)	595	- **0.172(** < **0.001)**	595	**0.083(0.042)**	595	**0.093(0.023)**	595	**0.101(0.013)**	595	0.043(0.295)	595	0.026(0.533)
Acetone (mmol L^−1^)	595	- 0.058(0.156)	595	**0.144(0.0004)**	595	- 0.002(0.968)	595	- 0.0240(0.567)	595	- **0.240(** < **0.001)**	595	- 0.070(0.086)
BHB (mmol L^−1^)	595	- 0.028(0.496)	595	**0.263(** < **0.001)**	595	**0.159(** < **0.001)**	595	**0.101(0.014)**	595	- **0.231(** < **0.001)**	595	- **0.255(** < **0.001)**
FFAs (mmol 100 g^−1^ fat)	582	**0.257(** < **0.001)**	582	0.014(0.741)	582	0.010(0.816)	582	0.043(0.301)	582	0.055(0.188)	582	**0.105(0.011)**
SCS (log)	671	- **0.095(0.014)**	671	-0.012(0.749)	671	0.062(0.108)	671	0.003(0.940)	671	0.018(0.633)	671	- **0.277(** < **0.001)**
Fatty acids (g 100 g^−1^ milk)												
Palmitic acid	595	- **0.256(** < **0.001)**	595	**0.571(** < **0.001)**	595	**0.814(** < **0.001)**	595	**0.850(** < **0.001)**	595	**0.491(** < **0.001)**	595	- 0.030(0.460)
Stearic acid	595	- **0.179(** < **0.001)**	595	**0.699(** < **0.001)**	595	**0.724(** < **0.001)**	595	**0.758(** < **0.001)**	595	**0.174(** < **0.001)**	595	- 0.008(0.839)
Oleic acid	595	- **0.158(0.0001)**	595	**0.718(** < **0.001)**	595	**0.745(** < **0.001)**	595	**0.756(** < **0.001)**	595	**0.1387(0.001)**	595	- **0.106(0.009)**
Short-chain FAs	597	- **0.088(0.031)**	597	**0.636(** < **0.001)**	597	**0.862(** < **0.001)**	597	**0.903(** < **0.001)**	597	**0.490(** < **0.001)**	597	- 0.020(0.623)
Medium-chain FAs	595	- **0.332(** < **0.001)**	595	**0.475(** < **0.001)**	595	**0.823(** < **0.001)**	595	**0.799(** < **0.001)**	595	**0.563(** < **0.001)**	595	- **0.274(** < **0.001)**
Long-chain FAs	595	- **0.165(** < **0.001)**	595	**0.739(** < **0.001)**	595	**0.748(** < **0.001)**	595	**0.769(** < **0.001)**	595	**0.130(0.002)**	595	- 0.0717(0.085)
Saturated FAs	597	- **0.215(** < **0.001)**	597	**0.649(** < **0.001)**	597	**0.884(** < **0.001)**	597	**0.915(** < **0.001)**	597	**0.485(** < **0.001)**	597	- 0.068(0.096)
Monounsaturated FAs	595	- **0.172(** < **0.001)**	595	**0.692(** < **0.001)**	595	**0.757(** < **0.001)**	595	**0.779(** < **0.001)**	595	**0.216(** < **0.001)**	595	- 0.067(0.103)
Polyunsaturated FAs	595	- 0.060(0.142)	595	**0.419(** < **0.001)**	595	**0.529(** < **0.001)**	595	**0.595(** < **0.001)**	595	**0.341(** < **0.001)**	595	**0.163(** < **0.001)**
*Trans* FAs	597	**0.081(0.048)**	597	**0.383(** < **0.001)**	597	- 0.024(0.566)	597	**0.104(0.011)**	597	- **0.405(** < **0.001)**	597	**0.526(** < **0.001)**

	n	Urea (mmol L^−1^)	n	Citric acid (%)	n	Acetone (mmol L^−1^)	n	BHB (mmol L^−1^)	n	FFAs (mmol 100 g^−1^ fat)
Citric acid (%)	591	- **0.251(** < **0.001)**		–						
Acetone (mmol L^−1^)	591	- **0.153(0.001)**	595	**0.336(** < **0.001)**		–				
BHB (mmol L^−1^)	591	- **0.122(0.003)**	595	**0.298(** < **0.001)**	595	**0.810(** < **0.001)**		–		
FFAs (mmol 100 g^−1^ fat)	578	**0.263(** < **0.001)**	582	- **0.091(0.029)**	582	- **0.216(** < **0.001)**	582	- **0.196(** < **0.001)**		–
SCS (log)	548	- **0.104(0.015)**	545	- **0.114(0.008)**	545	**0.133(0.002)**	545	**0.207(** < **0.001)**	532	- **0.230(** < **0.001)**
Fatty acids (g 100 g^−1^ milk)										
Palmitic acid	593	- **0.089(0.031)**	590	**0.141(0.001)**	590	- 0.061(0.141)	590	0.033(0.426)	577	0.062(0.137)
Stearic acid	593	- 0.041(0.321)	590	**0.377(** < **0.001)**	590	**0.251(** < **0.001)**	590	**0.223(** < **0.001)**	577	- **0.139(0.001)**
Oleic acid	593	- 0.078(0.059)	590	**0.188(** < **0.001)**	590	**0.147(0.001)**	590	**0.173(** < **0.001)**	577	- **0.085(0.041)**
Short-chain FAs	595	**0.177(** < **0.001)**	592	- 0.004(0.920)	592	- **0.147(0.001)**	592	- **0.085(0.039)**	579	- 0.007(0.876)
Medium-chain FAs	593	- **0.126(0.002)**	590	**0.313(** < **0.001)**	590	0.053(0.202)	590	**0.094(0.023)**	577	- 0.059(0.160)
Long-chain FAs	593	- 0.056(0.173)	590	**0.266(** < **0.001)**	590	**0.234(** < **0.001)**	590	**0.234(** < **0.001)**	577	- **0.131(0.002)**
Saturated FAs	595	0.006(0.888)	592	**0.165(** < **0.001)**	592	- 0.037(0.368)	592	0.049(0.230)	579	0.013(0.746)
Monounsaturated FAs	593	- 0.075(0.068)	590	**0.193(** < **0.001)**	590	**0.167(** < **0.001)**	590	**0.177(** < **0.001)**	577	- **0.097(0.020)**
Polyunsaturated FAs	593	0.076(0.065)	590	0.006(0.878)	590	- **0.289(** < **0.001)**	590	- **0.291(** < **0.001)**	577	**0.091(0.029)**
*Trans* FAs	595	- 0.043(0.292)	592	**0.260(** < **0.001)**	592	0.049(0.234)	592	- 0.001(0.991)	579	- **0.114(0.006)**

n – number of samples; SCS – somatic cell score = log_10_(somatic cell count) + 3; BHB – β -hydroxybutyric acid; FFAs – free fatty acids; FAs – fatty acids.

In the present study, the effects of gene polymorphisms were generally limited, as were the interactions between gene and farm, gene and lactation stage, and parity and lactation stage. However, previous studies have reported significant associations with FA composition and have emphasized the relevance of the *FASN*, *SCD1*, and *DGAT1* genes for metabolic regulation and susceptibility to ketosis (Li et al., 2016; Nafikov et al., 2014; Su et al., 2023; Zhu et al., 2019). In agreement with the importance of candidate genes affecting milk composition, Ardicli et al. (2024) found that combined *SCD1* and *FABP4 *genotypes significantly affected milk fat content. Similarly, Safina et al. (2018) reported a significant influence of *SCD1* polymorphism on total milk yield and lactation persistence. Mauric et al. (2019) demonstrated an effect of *FASN* gene polymorphism on the proportions of saturated and monounsaturated FAs, particularly in Simmental cattle. When the BHB level was evaluated as an indicator of ketosis, a significant farm effect was observed in all models. In models including *AGPAT6*, *SCD1*, and *DGAT1* genes, the effect was also influenced by parity. In the model that included the *FASN* gene, the parity effect was near the significance threshold. The BHB content was 0.046 mmol L^−1^ in the first lactation, 0.062 mmol L^−1^ in the second lactation, and 0.057 mmol L^−1^ in the third lactation (data not given in the table). The difference in BHB content between the first and second lactations was significant at p<0.05 .

Interestingly, the influence of the lactation stage on the BHB content was not significant, in contrast to the effects for other milk components. However, the interactions between parity and lactation stage were significant in models including *AGPAT6*, *SCD1*, and *FASN* genes and were near the threshold of significance in the model including *DGAT1*.

For protein and lactose contents, significant effects were observed for parity and lactation stage and, in some cases, for the interactions of farm–lactation stage and farm–gene polymorphism. Levels of urea, citric acid, acetone, and FAs were influenced by the lactation stage, with the urea level also being affected by the farm–lactation stage interaction. The components mentioned were only rarely influenced by gene polymorphisms (Tables 2–6).

Phenotypic correlations among the milk components are given in Table 7. Daily milk production correlated negatively and most significantly with the contents of the evaluated parameters. A significant positive correlation was found between urea content and milk yield. These findings indicate a higher metabolic intensity in high-producing cows, in agreement with previous studies (Zhao et al., 2025). However, they also reported a positive correlation between urea and protein content, whereas we reported a very low, significantly negative correlation. A significant correlation between protein and fat contents (0.346; p<0.001 ) was observed.

The correlations associated with BHB concentration were of particular interest. BHB content was positively correlated with most FA contents, with only a few exceptions. The correlations between BHB content and the contents of protein, lactose, urea, and long-chain FAs were negative, similarly to the findings of Magro et al. (2025). This may suggest differences in the metabolism of the mentioned substances. The correlations between BHB content and the FPR, as well as between BHB content and the fat / lactose ratio, were significantly positive. These findings align with similar correlations observed for FAs; therefore, the correlations with protein, lactose, and urea levels were significantly negative. Similar results were reported by Benedet et al. (2019). A significant positive correlation between the BHB content and the FPR was also reported by Magro et al. (2025).

Other authors reported lower milk fat content, unchanged protein content, and a somewhat higher milk yield in hyperketonaemic cows compared with healthy cows, findings that are inconsistent with ours (Pothmann et al., 2026). However, those authors diagnosed ketosis based on blood BHB concentrations measured on days 7, 14, and 28 of lactation, whereas, in our study, milk composition and production traits were evaluated throughout the entire lactation period. In contrast, Kučević et al. (2026) found positive correlations between milk BHB concentration and both milk fat content and FPR, as well as a negative correlation with lactose content, which is in agreement with our results (Kučević et al., 2026).

A very strong correlation (0.810; p<0.001 ) was observed between BHB and acetone (Table 8). This reflects their metabolism, particularly during the NEB period, when there is a greater release of free FAs into the blood. These are oxidized in the liver to ketone bodies, mainly BHB, acetoacetate, and acetone. Ketones enter the blood, urine, and milk (Bezdíček et al., 2024). Reus et al. (2023) reported significant associations and interactions between cow types (based on BHB and NEFA concentrations and the FPR); parity; lactation stage; milk yield; and the proportions of C16:0, C18:0, and C18:1, suggesting opportunities for health screening (Roy et al., 2006). Additionally, other authors suggest using the FPR to identify hyperketonaemia in dairy cows (Antanaitis et al., 2023). Others reported strong associations between the FPR and blood BHB. However, the coefficients of determination were very low, and no FPR threshold provided acceptable sensitivity and specificity for identifying hyperketonaemia (King et al., 2019). Although the FPR can indicate NEB at the herd level, it cannot accurately predict NEB at the individual cow level (Cabezas-Garcia et al., 2021). Further research is needed to develop reliable and practical methods for identifying cows at risk of hyperketonaemia and healthy animals under field conditions (Benedet et al., 2019). Magro et al. (2025) reported a genetic correlation of 0.45 between blood and milk BHB, suggesting the potential use of milk BHB levels assessed during milk performance testing for field detection of cows with ketosis.

The significant negative correlation ( - 0.277; p<0.001 ) between lactose content and the SCS confirms lactose's role as an indicator of the mammary gland's health status, consistent with the findings reported by Satoła (2025). The authors reported positive associations between the SCS and protein and fat contents, as well as the FPR, which is in accordance with our results. We found a weak but statistically significant correlation (0.207; p<0.001 ) between the SCS and BHB content. The nonsignificant correlation ( - 0.104) between the SCS and the urea content in our analysis is in agreement with Dreyer et al. (2025). They concluded that milk urea content is influenced by a wide variety of factors, including the cow's health status, and should not be used as an indicator for an individual cow but rather as a tool for evaluating the nutritional status of the protein supply in a herd. Potential shifts should be considered for herds with a high prevalence of sick cows (Dreyer et al., 2025). Even the bulk tank milk analysis of urea and protein content provides indications of herd nutrition (Lean and Golder, 2024). Approaches based on automatically recorded behavioural and milk composition data, combined with machine learning algorithms, may facilitate the early detection of metabolic and udder health disorders in dairy cows (Girdauskaitė et al., 2025).

One limitation of the present study is that the analysed animals originated from a limited number of farms. In addition, although most genotypes were represented by adequate numbers of animals, some genotypes occurred only rarely. In particular, the AA genotype of the FASN polymorphism was detected in only two animals, which may have reduced the statistical power to detect genotype effects. Therefore, the results associated with this genotype should be interpreted with caution and verified in larger populations.

## 4 Conclusions

For milk composition, farms had a strong effect, which reflects the importance of housing; treatment; milking; and, in particular, differences in feeding ratios. An important factor is also the lactation stage, which relates to changes in daily milk yield and is linked to changes in component contents, i.e. the persistence of the lactation curve. Observed polymorphisms of lipogenic genes had a low effect on both milk parameters and the fatty acid profile. The association between β -hydroxybutyrate and the fat-to-protein ratio was significant, but the prediction of ketosis in individual cows does not seem to be reliable. The fat-to-protein ratio may serve as an indicator of negative energy balance at the herd level but not at the individual cow level. The implementation of automatic milk composition recording, including β -hydroxybutyrate measurements, may offer valuable opportunities to improve metabolic monitoring in dairy herds in the future.

## Supplement

10.5194/aab-69-541-2026-supplement The supplement related to this article is available online at https://doi.org/10.5194/aab-69-541-2026-supplement.

## Data Availability

The datasets are very extensive and highly complex; therefore, they are available from the corresponding author upon request.
