# Supplementary material for: Genetic and non-genetic factors influencing milk composition and cow metabolism
Source: Arch Anim Breed. 2026 Oct 2;69(4):541–52. doi: 10.5194/aab-69-541-2026 (PMC13632543; doi:10.5194/aab-69-541-2026)
Supplement: Supplementary file 1 [file aab-69-541-2026-supplement.pdf]

Supplement of Arch. Anim. Breed., 69, 541–552, 2026
https://doi.org/10.5194/aab-69-541-2026-supplement
© Author(s) 2026. CC BY 4.0 License.

Open Access
Archives Animal Breeding

Supplement of

Genetic and non-genetic factors influencing milk composition and cow metabolism

Jindřich Čítek et al.

Correspondence to: Eva Samková (samkova@fzt.jcu.cz)

The copyright of individual parts of the supplement might differ from the article licence.

Supplementary Material:

Title: Genetic and nongenetic factors influencing milk composition and cow metabolism (Čítek, J. et a1.)

S1. Genotyping

AGPAT6 g.36198118GGC genotyping was performed using primers AGPAT6_F (5´-FAM-CAAGGC GGCGTAGACAAA-3´) and AGPAT6_R (5´-AGCCCCGTCAGAGGTTCAT-3´). PCR analyses were carried out in the total volume 10 µl containing 1xPPP Master Mix (Top-Bio), 12.5 pmol of each primer, 10 ng of template DNA. Amplification was performed in TAdvance Thermal Cycler (Analytik Jena, Germany) using the following programme: pre-denaturation for 5 min at 95°C, 35 cycles 45 s at 95°C, 45 s at 60°C and 45 s at 72°C, finally, 10 min at 72°C. The *AGPAT6* gene polymorphism was tested on an Applied Biosystems ABI® 3500 DX Genetic Analyser (8-capillary sequencer) using the G5 dye set and POP7 polymer with a 50-cm capillary length (Thermo Fisher Scientific,USA). The results of the fragmentation analysis were processed using GeneMapper software (Thermo Fisher Scientific,USA), and the genotypes of the *AGPAT6* gene were determined. The output of the fragmentation analysis revealed distinct alleles differing in length, i.e., the polymorphism indel at BTA27 g.36198118GGC. Homozygous individuals had a product size of 174 or 177 bp (in/del GGC), while heterozygous individuals had both 174 and 177 bp alleles; presented in Littlejohn et al. (2014).

SCD1 gene alleles (c.878T>C) genotyping was performed using the primers SCD_F (5`-ACCTGGCTGGTGAATAGTGCT-3´) and SCD_R (5´-TCTGGCACGTAACCTAATACCCT-3´). PCR analyses were carried out in the total volume 25 µl containing 1xPPP Master Mix (Top-Bio), 12.5 pmol of each primer, 10 ng of template DNA. Amplification was performed in TAdvance Thermal Cycler (Analytik Jena, Germany) using the following programme: pre-denaturation for 5 min at 95°C, 30 cycles 20 s at 95°C, 20 s at 61°C and 30 s at 72°C, finally, 10 min at 72°C. PCR products were digested by the restriction enzyme *Fnu*4HI (5´-GC↓NGC-3´) (NEB, USA) overnight at 37°C. The TT genotype produced fragments 122, 28 and 20 bp, genotype TC produced fragments 122, 75, 47, 28 and 20 bp, and CC genotype produced fragments 75, 47, 28 and 20 bp (Inostroza et al. 2013).

FASN gene alleles g.17924A>G SNP genotyping was performed using the primers FASN_F (5'-TGCTGTCCATGCGGGAAATC-3') and FASN_R (5'-TGAGCACATCTCGAAAGCCA-3'). PCR analyses were carried out in the total volume 25 µl containing 1xPPP Master Mix (Top-Bio), 12.5 pmol of each primer, 10 ng of template DNA. Amplification was performed in TAdvance Thermal Cycler (Analytik Jena, Germany) using the following programme: pre-denaturation for 2 min at 95°C, 30 cycles 20 s at 95°C, 20 s at 56°C and 20 s at 72°C, finally, 10 min at 72°C. PCR products were digested by the restriction enzyme *Msc*I (5'-TGG↓CCA-3′) (NEB, USA) at 37°C overnight. PCR total amplicon was 759 bp. The resulting AA genotype produced three fragments of 345, 251 and 166 bp, the AG genotype produced four fragments of 417, 342, 251 and 166 bp, the GG genotype produced two fragments of 417 and 342 bp. Described in Roy et al. (2006); Cancino-Baier et al. (2021); Inostroza et al. (2013).

LEP gene alleles M and W were genotyped using the primers LEP_F (5´GGGAAGGGCAGAAAGATAG) and LEP_R (3´TGGCAGACTGTTGAGGATC). PCR analyses were carried out in the total volume 25 µl containing

1xPPP Master Mix (Top-Bio), 12.5 pmol of each primer, 10 ng of template DNA. Amplification was performed in TAdvance Thermal Cycler (Analytik Jena, Germany) using the following programme: pre-denaturation for 10 min at 94°C, 30 cycles 30 s at 94°C, 30 s at 53°C and 60 s at 72°C, finally, 10 min at 72°C. PCR products were digested by the restriction enzyme *Hph*I (5'-GGTGA NNNNNNN↓-3′) (NEB, USA) at 37°C overnight. The resulting fragment allele M one fragment of 331 bp, allele W two fragments, 311 bp and 20 bp, as described by Buchanan et al. (2002); Haegeman et al. (2000).

DGAT1 gene alleles A (alanine) and K (lysine) the GC → AA exchange at positions 10433/10434; genotyped using the primers DGAT1_F (5′-GCACCATCCTCTTCCTCAAG) and DGAT1_R (3′-GGGGGCGAAGAGGAAGTAGTAG). PCR analyses were carried out in the total volume 25 µl containing 1xPPP Master Mix (Top-Bio), 12.5 pmol of each primer, 10 ng of template DNA. Amplification was performed in TAdvance Thermal Cycler (Analytik Jena, Germany) using the following programme: pre-denaturation for 5 min at 95°C, 35 cycles 45 s at 94°C, 45 s at 61°C and 45 s at 72°C, finally, 5 min at 72°C. PCR products were digested by the restriction enzyme *Cfr*10I (5′-Py↓GGCCPu-3′) (ThermoFisher, USA) at 37°C overnight. The resulting fragment allele A one fragment of 411 bp, allele K two fragments, 208 bp and 203 bp. Described in Kühn et al. (2004).

All results from the PCR-RFLP analyses were visualized on a 2% agarose gel using EtBr staining, imaged using an InGenius 3 imaging system, and analyzed using GeneSys Software (Syngene, UK).

Table S1: Genotype and allele frequencies of the gene in the combined group of Holstein and Czech Simmental cows

Gene	Genotype	n	(%)	χ^2	Allele frequencies	
	KK	0	0		*K*	*L*
DGAT1	*KA*	25	5.58	0.369^{ns}		
	AA	423	94.42		0.03	0.97
	MM	290	75.72		*M*	*W*
LEP	*MW*	77	20.10	11.915***		
	WW	16	4.18		0.86	0.14
	TT	52	11.63		*T*	*C*
SCD1	*TC*	263	58.84	20.792***		
	CC	132	29.53		0.41	0.59
	AA	2	0.45		*A*	*G*
FASN	*AG*	116	25.95	6.071*		
	GG	329	73.60		0.13	0.87
	CC	122	28.11	107.571***	*C*	*T*
AGPAT6	*CT*	303	69.82			
	TT	9	2.07		0.63	0.37

χ2 – chi-square statistic for the Hardy-Weinberg equilibrium (HWE) test; *($p < 0.05$); ***($p < 0.001$), ns: non-significant, the group was in HWE
